# LEXpander: Applying colexification networks to automated lexicon expansion

**DOI:** 10.3758/s13428-023-02063-y

**Published:** 2023-03-10

**Authors:** Anna Di Natale, David Garcia

**Affiliations:** 1 Institute of Interactive Systems and Data Science, Graz University of Technology, Inffeldgasse 16c/I, Graz, 8010 Austria; 2 Section for Science of Complex Systems, Medical University of Vienna, Spitalgasse 23, 1090 Vienna, Austria; 3 Complexity Science Hub Vienna, Josefstädter Straße 39, 1080 Vienna, Austria; 4 Department of Politics and Public Administration, University of Konstanz, Universitätsstraße 10, 78464 Konstanz, Germany

**Keywords:** Colexification networks, Lexicon expansion, Text analysis, Word embeddings

## Abstract

Recent approaches to text analysis from social media and other corpora rely on word lists to detect topics, measure meaning, or to select relevant documents. These lists are often generated by applying computational lexicon expansion methods to small, manually curated sets of seed words. Despite the wide use of this approach, we still lack an exhaustive comparative analysis of the performance of lexicon expansion methods and how they can be improved with additional linguistic data. In this work, we present LEXpander, a method for lexicon expansion that leverages novel data on colexification, i.e., semantic networks connecting words with multiple meanings according to shared senses. We evaluate LEXpander in a benchmark including widely used methods for lexicon expansion based on word embedding models and synonym networks. We find that LEXpander outperforms existing approaches in terms of both precision and the trade-off between precision and recall of generated word lists in a variety of tests. Our benchmark includes several linguistic categories, as words relating to the financial area or to the concept of friendship, and sentiment variables in English and German. We also show that the expanded word lists constitute a high-performing text analysis method in application cases to various English corpora. This way, LEXpander poses a systematic automated solution to expand short lists of words into exhaustive and accurate word lists that can closely approximate word lists generated by experts in psychology and linguistics.

**Supplementary Information:**

The online version contains supplementary material available at 10.3758/s13428-023-02063-y.

## Introduction

Lists of words are widely used in many text analysis and NLP tasks, either in a pre-processing step to retrieve relevant instances in texts or as a necessary part of text analysis algorithms, for example in methods relying on word counts. Even when applying methods not based on word lists, for example neural networks, the selection of the texts to retrieve and analyze is often based on some thematic word lists to query in larger corpora. For example, in order to classify suicide-related tweets with machine learning methods, Metzler, Baginski, Niederkrotenthaler and Garcia (2022) first retrieve relevant tweets on the basis of a word list. Furthermore, established benchmarks in text analysis, such as the SemEval benchmark for sentiment analysis (Rosenthal, Farra, & Nakov, 2017) and the Tweeteval benchmark (Barbieri, Camacho-Collados, Espinosa Anke, & Neves, 2020), are based on querying large text sources (e.g., the whole of Twitter) by using pre-specified word lists.

In this work, we focus on word lists dealing with a subject, i.e., categorical lexica. These word lists, or lexica, are related to a chosen topic or behavior but do not have additional variables or metadata such as valence or sentiment ratings which indicate the strength of the belonging to a certain category. Categorical lexica are either created from scratch or adapted from already published word lists used in previous studies. Already-existing word lists might be adapted to research questions which are slightly different from the original ones. In many cases, the novel setting or the research questions of a new study require a modification of the original word list. These modifications can range from the exclusion of words not suitable for the topic analyzed, as in Metzler et al., (2022) and Jaidka et al., (2020), to the translation of those word lists into other languages, as in Werlen, Imhof and Bergamin, (2021). However, some of these manipulations could result in the introduction of noise or error in the new version of the word list. Indeed, modifications like the translation of expressions and words in a different language might not convey the same meaning as the original ones (Mohammad, 2020). Additionally, novel topics have to be addressed with word lists created ad hoc, as for example coronavirus-related word lists at the beginning of the COVID-19 pandemic, as in Banda et al., (2021), or word lists used to explore modes of drug administration not previously known to the medical personnel (Balsamo, Bajardi, Salomone, & Schifanella, 2021). Extended word lists can be manually created starting from a short selection of seed words and expanding them to create a final word list via brainstorming or with the use of a thesaurus. This approach often proves to be resource-intensive and prone to inconsistent results when replicated by different groups of people (King, Lam, & Roberts, 2017).

An alternative to manual lexicon expansion is the application of automated methods to find new words. One of the first approaches is the retrieval of synonyms and related words using semantic resources like WordNet (Miller, 1995). A more recent approach consists of the use of word embedding spaces to select the closest words to each seed word, as in Balsamo et al., (2021). Indeed, according to the distributional hypothesis (Firth, 1957), the retrieved words are semantically related to the chosen seed words. One of the most elaborated lexicon expansion methods is Empath (Fast, Chen, & Bernstein, 2016a), which deploys word embeddings to generate word lists from a list of seed words. In particular, Empath constructs the expanded word list considering the closest words to the cumulative vector of the seed words in the word embedding space. Although Empath is widely used in many studies (Ribeiro, Calais, Santos, Almeida, & Meira, 2018; Shing et al.,, 2018; Zirikly, Resnik, Uzuner, & Hollingshead, 2019), the method deploys an outdated word embedding model, as the algorithm has not been updated since its release.

Multiple solutions have been provided to solve the problem of lexicon expansion but researchers lack a systematic comparison of existing methods that allow both to find the best-performing ones and to validate their performance in relevant application scenarios. A notable resource in this extent is Lexifield (Mpouli, Beigbeder, & Largeron, 2020), a lexicon expansion algorithm that was compared to previous existing methods. While informative, the work on Lexifield is based on a narrow set of methods (word embedding-based and knowledge-based approaches) and on a limited set of topics (sound, taste, and odor). Another effort towards the creation of a baseline is constituted by Bozarth and Budak (2022). There, the authors investigate the problem in relation to the retrieval of tweets. In this article, we aim to provide a wider benchmark that includes methods based on synonym networks, word embeddings, and colexification networks. These methods will be used to expand word lists of wide use such as emotion words, sentiment words, words for behaviors (for example cognition and social interaction), as well as words for several other topics including family, religion, death, work and leisure.

Beyond a benchmark for lexicon expansion, we also present a novel approach to the problem of automatic lexicon expansion: LEXpander. LEXpander is based on a multilingual semantic network of cross-linguistic colexifications. Colexification is a linguistic phenomenon that occurs when two different concepts are conveyed using the same word in one language. This language is said to colexify the two concepts. For example, the two concepts ‘medicine’ and ‘poison’ are expressed with only one word, ‘pharmacon’, in Ancient Greek. Therefore, Ancient Greek colexifies the concepts of ‘poison’ and ‘medicine’. Since its coinage, the concept of colexification has been related to semantic similarity (François, 2008), that is concepts that are linked by colexification share semantic meaning (Karjus, Blythe, Kirby, Wang, & Smith, 2021; Xu, Duong, Malt, Malt, & Srinivasan, 2020).

Colexification occurrences have been collected from multiple linguistic resources and organized in a network structure, where concepts that are colexified by a number of languages are linked (Croft, 2022; List, Mayer, Terhalle, & Urban, 2014). The most known cross-linguistic colexification network is Clics^3^ (Rzymski et al., 2020) and is built from a wide range of linguistic resources. However, this network presents a small set of concepts (less than 2000 in the newest version), unsuitable for the application to lexicon expansion. In order to increase the language coverage of Clics^3^, colexification networks automatically built from bilingual dictionaries have been proposed in our previous work (Di Natale, Pellert, & Garcia, 2021). In Di Natale et al., (2021), we showed that these automatically built networks encode affective relationships and reach high performance when inferring the affective ratings of words, with the FreeDict colexification network being one of the most comprehensive.

In this paper, we adapt the inference method based on the FreeDict colexification network from Di Natale et al., (2021) to the expansion of categorical lexica, a method we call LEXpander. We use FreeDict instead of other colexification networks because FreeDict has been shown to yield to the best performance when recovering the affective meaning of words (Di Natale et al., 2021) and because it encompasses the highest number of words, nearly 28,000. In contrast to Di Natale et al., (2021), in this work we not only focus on the affective dimension of meaning but also aim at developing a tool of lexicon expansion, which can be applied to the most various themes. Furthermore, we do not tackle the problem of the inference of ratings of words. On the contrary, we aim at expanding thematic word lists, that is at selecting words related to a specific theme without specifying the intensity of this relationship.

The term colexification encompasses the two linguistic phenomena of polysemy and homonymy. While a polysemic word is defined as a word with multiple related meanings, homonyms are words with multiple senses which are not related. In order to deploy the definition of polysemy, that is the fact that meaning linked in the colexification network are related, it is necessary to filter out homonyms. As suggested by List et al., (2018), a practical solution for this problem is to filter the network according to the number or languages and/or families of languages that present the same colexification pattern. Indeed, this filtering clears the network from most spurious links, as they would appear in only one or a few records. In Clics^3^, the authors suggest including colexifications that occur in at least three languages and three families of languages (Rzymski et al., 2020). Since FreeDict has a lower coverage of languages and families of languages, we exclude only occurrences that appear in less than two languages, as also indicated in Di Natale et al., (2021).

A novel feature of LEXpander is that, by design, it provides a multilingual solution to the problem of lexicon expansion. Indeed, the underlying structure of colexification networks is supra-lingual (Khishigsuren et al., 2022), that is it consists of concepts that go beyond single languages. This makes the system applicable to any language included in the translation data used to build the colexification network. Note that the supra-lingual feature of this method does not imply that its performance is independent from the language chosen. Rather, it means that the method can be applied to different languages without needing to adapt the framework to the specific language chosen. In this paper, we showcase this feature by testing LEXpander for the automatic expansion of lexica both in English and German. In the literature there are few attempts at expanding word lists in languages different from English. In particular, Mpouli et al., (2020) expand word lists in English and French, Zeng, Yang, Tu, Liu and Sun (2018) deploy Chinese linguistic resources and Thavareesan and Mahesan (2020) consider the expansion of sentiment-related word lists in Tamil. However, German is a language which has not yet been addressed in previous literature of lexicon expansion algorithms.

The contributions of this paper are the following:

- We propose a novel lexicon expansion method, LEXpander, which is based on a linguistic concept;
- We compare LEXpander with other widely used lexicon expansion algorithms establishing a benchmark for lexicon expansion algorithms;
- We show that LEXpander achieves the best precision and *F*_1_ (the trade-off between precision and recall) when expanding word lists in English and in German;
- We show that LEXpander has either the best or is tied with the best method in terms of correlation with exhaustive manual word lists in the analysis of English texts of online and traditional communication;
- We present an interactive web app to allow the easy use and extension of LEXpander.

## Methods

### Lexicon expansion algorithms

In this paper, we propose a novel method for lexicon expansion, LEXpander, and compare its performance with other approaches. Text analysis applications often rely on the ad hoc creation of lexica which ideally collect all the words used to refer to a topic. LEXpander automatizes the task of creating such word lists starting from a small set of seed words. LEXpander is based on a colexification network, that is a multilingual semantic network whose structure is supra-lingual.

The LEXpander model is built as follows. Given the adjacency matrix of the colexification network *A* = {*A*_*ij*_}, such that:

$$ A_{ij}=\begin{cases} 1 & \text{if concepts}~i~\text{and}~j~\text{are colexified by at least two languages}\\ 0 & \text{otherwise} \end{cases} $$

and *S* = {*s*} set of seed words, the expanded lexicon is defined as *L* = *S* ∪ *W*, where:

1 $$ W=\left\{ w \mid A_{sw}=1, \forall s \in S \right\}  $$

**Fig. 1:**
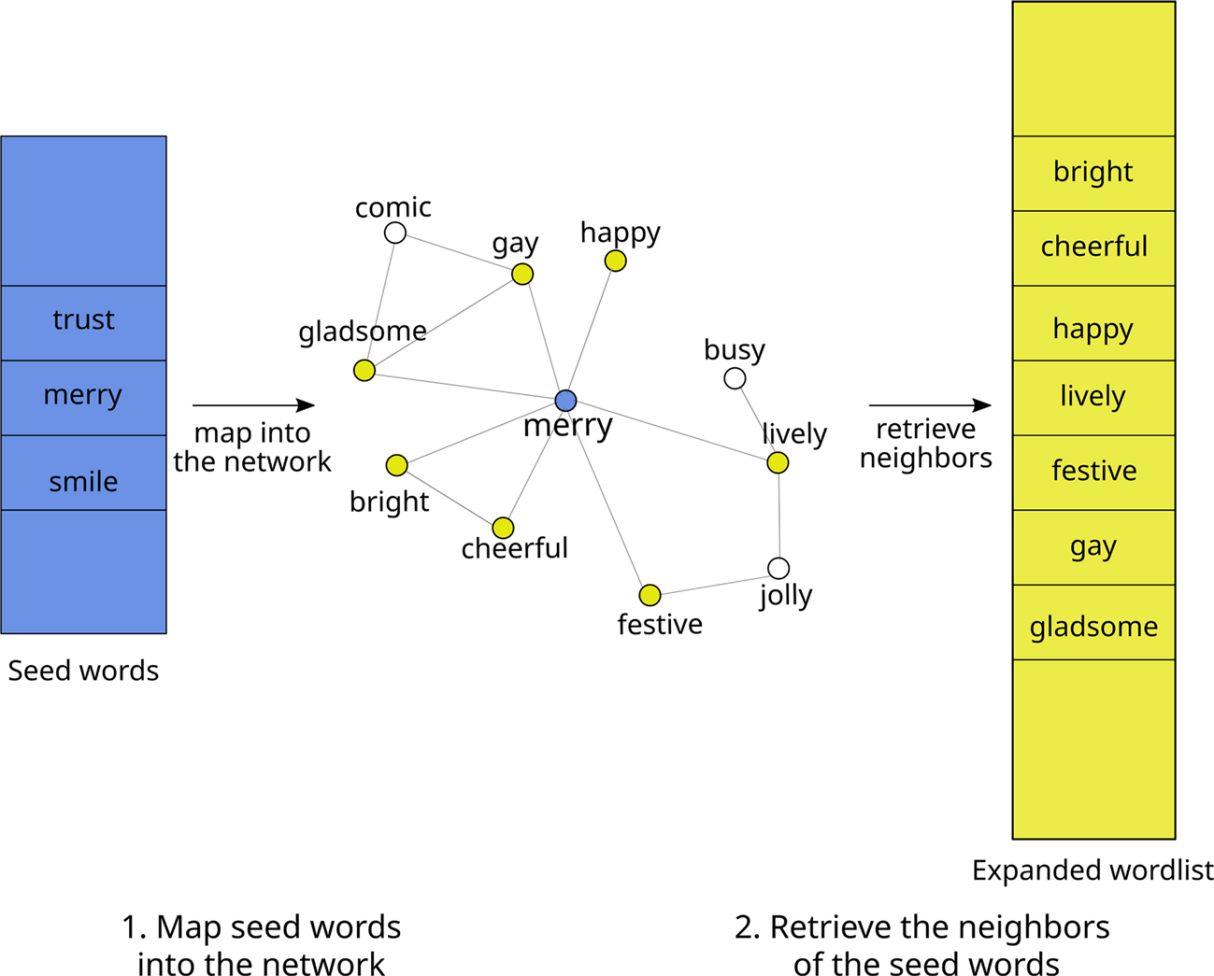
Representation of the LEXpander algorithm. Seed words (*blue*, *on the left*) are mapped onto the network (step 1) and the neighbors of those words (*yellow*, *on the right*) are retrieved to create the expanded word list (step 2). In the figure, we only represent the process in the case of one word, ‘merry’ and we label the nodes in the colexification network according to their English word

In other words, given a set of seed words *S*, LEXpander creates a longer lexicon by retrieving all the neighbors of the seed words in the colexification network, as represented in Fig. 1.

Note that the method introduced in Eq. 1 hypothetically works for any language. This is a consequence of the supra-lingual feature of the colexification network. When expanding word lists in English, we pick the English word for each concept in the network, while when considering German we do the same by selecting the German words that convey the selected concepts. In order to expand word lists in German, we deploy Eq. 1 with the German version of the colexification network. The German version of the network is obtained by selecting the German word for each supra-lingual concept of the network.

Links in colexification networks can be weighted by the number of languages and the number of families of languages that colexify the same pair of concepts (List et al., 2018). These weights can be used to remove spurious patterns that would contribute to noise in the network and to estimate the strength of the relationship between concepts. While we do use weights to select meaningful links in the network, we do not consider the link weights when expanding word lists. Indeed, link weights give an estimate of the intensity of the relationship between words, which was for example used in Di Natale et al., (2021) to infer the affective ratings of words. On the contrary, the present study deals with categorical lexica and LEXpander needs to identify words that belong to a chosen topic, not the intensity of the relationship with such topic. Therefore, LEXpander is based on a filtered, unweighted colexification network.

We compare the performance of LEXpander with various other automatic lexicon expansion algorithms. These methods can be divided in two classes: methods based on semantic networks and methods which deploy word embeddings. As methods based on semantic networks, we consider the widely used network of synsets retrieved from WordNet (Miller, 1995) for the expansion of English word lists and an open source version of WordNet in German, OdeNet (Siegel and Bond, 2021). The lexicon expansion approach which deploys these semantic networks is similar to the one used for LEXpander in Eq. 1: the expansion of a set of seed words consists in the retrieval of the neighbors of all the seed words in the network. One necessary step of the expansion of lexica using semantic networks is the mapping of the seed words onto the network (see Fig. 1, step 1). However, in some cases the seed words cannot be mapped onto the network and the expansion of the word list is not possible. In this case, the result of the expansion is an empty word list. Thus, together with the performance of each method we also consider the number of word lists the method could expand as a way to consider this case.^1^

A second class of methods we consider are approaches based on word embeddings. The creation of word embeddings involves the usage of neural models which are trained on textual data. In particular, we consider methods based on the GloVe model trained on the English Wikipedia (Pennington, Socher, & Manning, 2014) and on the German Wikipedia and the FastText model trained on the English Wikipedia and on the German Wikipedia using the skipgram model (Bojanowski, Grave, Joulin, & Mikolov, 2017). The pretrained vectors for FastText were obtained from https://fasttext.cc/, while the English GloVe word embedding was retrieved with the R package text2vec (Selivanov, Bickel, & Wang, 2020). The pretrained vectors for the German GloVe was obtained from https://www.deepset.ai/german-word-embeddings. We consider the 25,000 most used words according to Google books (https://books.google.com/ngrams/) in the application of word embedding spaces to lexicon expansion.

We implement the word list expansion from word embedding spaces following the method of Mpouli et al., (2020). The first method retrieves all the words in a word embedding model that have a cosine similarity above a threshold that has been calibrated in previous work (Mpouli et al., 2020). Furthermore, we consider one more elaborated method based on word embeddings: Empath (Fast et al., 2016a). This algorithm creates the extended word list by retrieving the closest words to the embedding of the cumulative vector relative to the seed words. We deploy this method via the Empath Python package (Fast, Chen, & Bernstein, 2016b) with the default size setting, which is set to an output of 100 words for the expanded word list. Since the number of words was too low for our purposes, we tried to increase this value and considered size 300, 500, 700, and 1000. However, with these specification the Python package for Empath gave an error and did not deliver any output. Moreover, Empath is based on an old word embedding model which has not been updated since its first release. As a consequence, we decided to implement a novel version of the method and consider only that in the analyses.

In particular, we re-implemented the Empath method using the newer FastText word embedding space trained on the English and German Wikipedia. Thus, we obtain two new versions of Empath, one for the expansion of English lexica and one for the expansion of German lexica. We call these re-implementations Empath 2.0. Note that, while Empath allows for the selection of the size of the final word list, Empath 2.0 does not have this feature because the mechanism used in Empath for discarding words was not documented in the original paper. As a consequence, the sizes of the word lists computed by the two versions of Empath differ, but we consider only Empath 2.0 as it is based on newer and more exhaustive word embedding models.

For each of the methods, we also define a random baseline method based on the length of the resulting word lists. In the baseline algorithm, we perform 1000 random samples of words from the relative networks or word embedding spaces of the same size of the expanded lexicon resulting from each method. This serves as a null model to measure what would be the performance of a random guess when expanding word lists to given sizes.

### Empirical analysis

In this section, we introduce the framework for the comparison of the lexica expansion algorithm and describe the tests we perform for the evaluation of said algorithms. The framework here presented can be easily applied in future research to provide a state-of-the-art approach to word list expansion and to improve the replicability and validity of future text analysis.

#### Empirical framework

We test the performance of the lexicon expansion methods in expanding sets of seed words selected from the 2015 English version (Pennebaker, Boyd, Jordan, & Blackburn, 2015) and the 2007 German version of the Linguistic Inquiry and Word Count (LIWC) (Wolf et al., 2008). LIWC is a widely used proprietary dictionary-based method for the analysis of texts (Pennebaker, Francis, & Booth, 2001). The 2015 English version of LIWC collects 73 word lists relative to various topics, including for example words indicating future orientation or referring to the family sphere. Such word lists have been used in many influential studies, as for example (Kleinberg, van der Vegt, & Mozes, 2020; Shing et al.,, 2018; Zirikly et al.,, 2019). The popularity of LIWC resulted also in its translation in various languages. In particular, we consider the German version of LIWC from 2007, which collects word lists belonging to 68 different topics.

Recently, a new version of LIWC has been released (Boyd, Ashokkumar, Seraj, & Pennebaker, 2022). In this new version, new categories have been added, while some classes from previous versions have been merged together. Since we use LIWC only as a means to test the performance of lexicon expansion algorithms, we do not think that the results of the paper would vastly change using the new version of LIWC. Indeed, this paper does not provide any statement about LIWC itself, rather it is a comparison between methods in a framework that takes into account LIWC word lists and only the relative performance of the methods analyzed here is important. Indeed, the single results of the methods in the various experiments when testing them on different versions of LIWC might change but the relative performances will not vary, that is the lexicon expansion methods that achieve the best performances in the framework used in this work would maintain the same ranking when tested in a framework that considers a novel version of LIWC.

Words in both the English version of LIWC from 2015 and the German from 2007 are given in a shortened form with wildcards (indicated with *) to indicate all the words with different endings but starting with the same sequence of letters. In a first preprocessing step, we match the words with wildcards with entries in dictionaries in order to retrieve the full-form words from the wildcard terms. For example, the word with wildcard ‘apprehens*’ from the English LIWC word list for negative emotion, is matched with the following entries in the dictionary: ‘apprehensible’, ‘apprehension’, ‘apprehensive’, ‘apprehensiveness’. We consider the lexica resulting from this matching procedure as the original LIWC word lists.

After the retrieval of the words in the LIWC word lists, the experiment is performed as follows: We first select a subset of words from each word list of LIWC to use as seed words in two ways, either at random or based on expert selection of shorter lexica called EVs, as published in Vine, Boyd and Pennebaker (2020). See “Precision study” for a more detailed explanation on EVs. We then input this seed word list in the expansion algorithms with the aim of recovering the original, complete LIWC word list. By doing so, we obtain expanded word lists. We then evaluate the performance of each lexicon expansion algorithm using three different tests.

### Evaluation

**Fig. 2:**
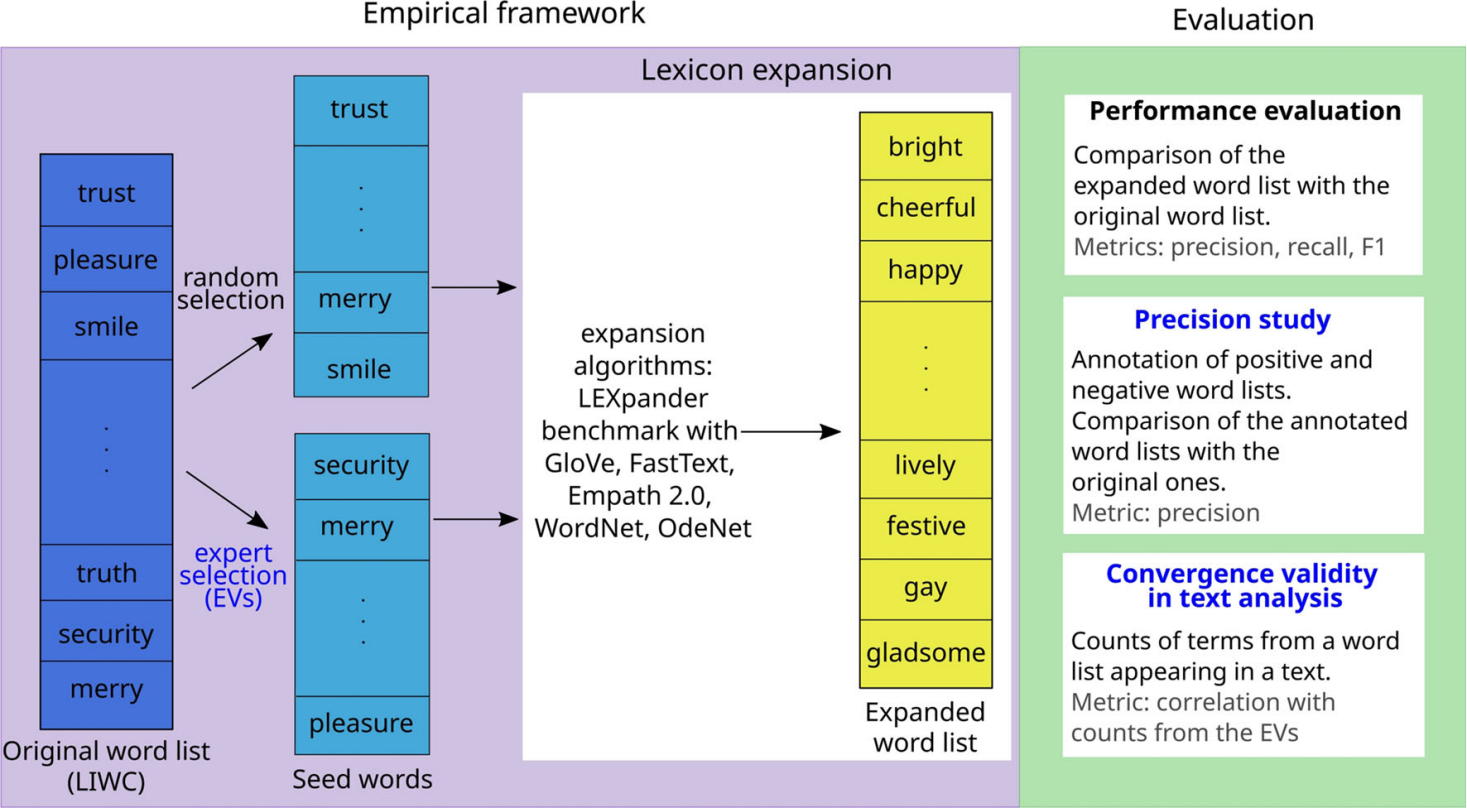
Representation of the pipeline used to evaluate the word list expansion methods. Seed words are selected from the original LIWC word list (*blue*, *on the left*) either at random or selected by experts. They are then used as input of the various expansion algorithms, which give an expanded word list as output (*yellow*, *in the center*). In this figure, we represent one example of such expanded word list. In order to assess the performance of each method, we perform three different evaluation tests. *Blue headlines* indicate steps and evaluations performed only in English

The evaluation of the lexicon expansion methods is performed according to three tests: the performance evaluation, the precision study and the convergence validity in text analysis. The performance evaluation will be performed both with English and German word lists, while the other two tests take into account only the English expansions. This is only due to the lack of resources in German, as all analyses could in principle be performed with resources in any language. A representation of such pipeline is depicted in Fig. 2. In the following sections, the tests are described in detail.

#### Performance evaluation

In this first task, we assess the performance of the methods in expanding various word lists given a set of seed words against longer lists generated by experts. In particular, given a set of seed words extracted from the thematic word lists of LIWC, we apply the lexicon expansion methods and retrieve the expanded word list. We then compare the expanded and original LIWC word lists and assess the performance of each method by computing the precision, recall and *F*_1_ of the method.

In particular, given $\tilde {L}$ original word list and *L* = *S* ∪ *W* expanded lexicon constructed as in Eq. 1, we define the true positives (TP) as the words of $\tilde {L}$ which are also present in *L* without seed words, that is in *W*. The false positives (FP) are the words in *W* that do not appear in $\tilde {L}$ and the false negatives (FN) are the words in $\tilde {L}$ not present in *W*. In other words:

2 $$ TP = \tilde{L} \cap W $$

3 $$ FP = W \setminus \tilde{L} $$

4 $$ FN = \tilde{L} \setminus W $$

We can define precision and recall as follows:

5 $$ precision = \frac{\mid TP \mid }{\mid TP \cup FP \mid } $$

6 $$ recall = \frac{ \mid TP \mid }{\mid TP \cup FN \mid} $$

*F*_1_ is the harmonic mean of the previous two quantities:

7 $$ F_{1} = 2 \frac{precision \cdot recall}{precision+recall} $$

The set of seed words is extracted from the thematic lexica of LIWC in two ways: either at random or selected by experts. The random selection of seed words from the LIWC lists is based on the selection of a percentage between 10% and 90% of the words of each word list, as depicted in Fig. 2. We repeat the experiment 50 times for each percentage, every time selecting a new random subset of seed words. We then compute precision, recall and *F*_1_ averaging on the 50 repetitions. The expert-based approach to the choice of the seed words is based on the words selected by the authors of Vine et al., (2020) as the most representative words for the LIWC categories of negative emotion, positive emotion, anxiety/fear, anger, sadness and undifferentiated negative emotion.^2^Vine et al., (2020) call these word lists Emotional Vocabularies (EVs). Note that the EVs are freely available online, therefore we openly redistribute the expanded word lists obtained from the EVs in our work. The EVs were not created as a set of seed words from which to recover the LIWC word lists. However, we use them as they are a freely available selection of words from LIWC. The EVs are available only in English, therefore we expand them only with the English lexicon expansion methods.

Note that the value of precision computed in the performance evaluation is a lower bound for the actual precision of the method: We only consider as successes the words that belong to the original LIWC word list. However, it can happen that the lexicon expansion method finds words which belong to the chosen topic but were not included in LIWC by the experts. Indeed, we do not consider the LIWC word lists to be exhaustive of the topic they deal with. As a consequence, in computing the precision, we consider as false positives some words which might actually be true cases. The estimate of the precision is thus a lower bound, since we cannot be completely certain that LIWC presents the most extensive word lists for each topic.

Furthermore, we compare the expansion of the random selection of words from LIWC and the expansion of the EVs. This comparison has the aim of testing whether the effort of manually selecting the most representative words of one class might be an advantage to the expansion of the word list. In order to do so, we consider the expansion of a random selection of seed words of the same length of the EVs from the five emotional categories. We repeat the random selection 50 times and consider the mean performance. We compare the results in terms of precision, recall and *F*_1_ with the performance of the expansion of the EVs.

Additionally, we analyze the interdependence of LEXpander with the other methods. In particular, we test whether the word lists created by the different methods capture different signals. In order to do so, we define a union and a intersection methods, which consist, respectively, of the union and intersection of the word lists resulting from the five expansion algorithms (LEXpander, WordNet, GloVe, FastText, Empath 2.0). We then analyze the performance of these methods when expanding the EVs in term of precision, recall and *F*_1_.

#### Precision study

As previously explained, the performance evaluation can only compute a lower bound for precision. To complement the analysis of lower bounds, we include an analysis of additional word annotations that do not appear in the LIWC words lists and can precisely assess the true value of precision. We call this test precision study. More in detail, we generated manual annotations of the word lists resulting from the expansion of the positive and negative EVs. Since the EVs are only available in English, this test can only be performed with English data. The first author and six other raters annotated the word lists. Five annotators are German native speakers, one speaks Italian and one Spanish as first language. They all have a near-native English proficiency. At least two annotators labeled each word in the expanded word lists and we select as relevant only the words which were accepted by both raters. In the case of FastText and Empath 2.0, the word lists resulting from the expansion procedure encompass more than 2000 words. In such cases, we annotate a random set of 300 positive and 300 negative words per method instead of the whole word list. In all other cases, all the words of the expanded word lists are annotated. In order to estimate the error of such statistic, we also compute the 95% confidence interval from the bootstrapping of the annotated word lists. The inter-rater agreement relative to the annotation of the positive words scores a Cohen’s *κ* of 0.59 (moderate agreement), while the task on the negative words achieves a Cohen’s *κ* of 0.65 (substantial agreement). Once the word lists have been annotated, we compute a more accurate estimate of the precision of each method.

#### Convergent validity in text analysis

We continue with a third task that compares the performance of the expanded lexica in an exercise of text analysis of English online communication and literary texts. In particular, we consider a simple text analysis method which consists in the computation of the frequency of words of the lexica expanded from the EVs and annotated which appear in the texts. We correlate the counts relative to each word list with the ones of the original lexica from LIWC on each single text snippet. We also compare the performance with the original EVs.

We compute the correlation of the annotated word lists obtained expanding the positive and negative EVs with the counts of LIWC on the texts of each single dataset. Note that the EVs are in English, therefore the text analysis exercise can be performed only with English texts. Since we only annotated the full-length word lists for LEXpander, WordNet and GloVe we can report the results relative to these methods. However, it is important to remember that the cleaning of the word lists was not carried out with a particular type of text in mind. This strategy would be the most advisable, but in the case of this paper we did not want to bias the results, therefore we use the same annotated word list for the four different types of texts.

For this analysis, we wanted to include texts of various lengths and from different sources in order to test whether the performances of the lexicon expansion methods depend on these features of the texts analyzed. In particular, we consider short texts of online communication from Reddit (in particular, all discussions, that is original post and answers, from the subreddits ‘antiwork’, ‘TwoXChromosomes’, ‘family’ and ‘Home’), longer texts from the Brown corpus (Francis and Kucera, 1979), texts collected in the Corpus of Historical American English (COHA; Davies, 2012) and all the tweets, excluding answers, published in the UK during one single day in February 2021. The number of documents and their average length is reported in Table 1. Thus, in this task we analyze the lexicon expansion methods against short and long texts from social media (respectively, Twitter and Reddit) and texts written in American English across a representative selection of works published in 1961 (Brown Corpus) and from a curated historical dataset that balances the genre of the texts with respect to their year of publication (COHA).

**Table 1:** Statistics of the datasets used for the text analysis exercise

Dataset	# Texts	Mean length
Brown corpus	502	2064
COHA	116,513	4852
Tweets	417,164	11
Reddit	54,499	1095

We report the number of texts in each dataset and their average length in number of words. Stop words are included in the counts

## Results

We report the results of the study comparing LEXpander, a lexicon expansion algorithm built on colexification networks, with other lexicon expansion methods.

### LIWC 2015 in English

In this section, we present the results of the evaluation tests relative to the 2015 version of LIWC in English.

#### Performance evaluation

In the first task, we use multiple lexicon expansion methods to retrieve the original LIWC word lists from a subset of their words. In Table 2 we report the results relative to a set of seed words chosen at random and amounting to 30% of the original LIWC word list. We also report the mean size of the expanded word lists and the results of the random baseline methods, averaged over 1000 repetitions. Additionally, the lengths of the expanded word lists for every experiment are featured in Tables 1, 2 and 3 of the Supplementary Materials.

**Table 2:** Results of the expansion of the random choice of 30% words from the English LIWC

Method	Precision		Recall		*F*_1_		Mean size
	Mean	bl	Mean	bl	Mean	bl	
LEXpander	**0.16**	0.01	0.14	0.02	**0.13**	0.01	614
WordNet^a^	0.10	0.00	0.07	0.00	0.07	0.00	525
Empath 2.0 ^b^	0.08	0.01	0.22	0.03	0.10	0.01	1,293
FastText ^c^	0.06	0.01	**0.29**	0.06	0.09	0.02	2,252
GloVe^d^	0.07	0.01	0.13	0.03	0.08	0.02	773

Precision, recall and *F*_1_ of the expansions generated from random 30% seed words compared to the original lexica from the English 2015 version of LIWC. Values are means computed over 50 samples of the seed words across word lists. We also report the mean length of the expanded lexica. The results of a random baseline method (bl) averaged on 1000 repetitions of the same length are also indicated. The best performances are highlighted in boldface

^a^ Miller (1995)

^b^ Bojanowski et al., (2017), Fast et al., (2016a)

^c^ Bojanowski et al., (2017)

^d^ Pennington et al., (2014)

Table 2 shows that the best precision and *F*_1_ scores are reached by LEXpander, while FastText yields to the highest recall when retrieving English word lists from a random selection of seed words. In this setting, LEXpander does not only achieve the best precision but also the best trade-off between precision and recall. Moreover, we observe that FastText and Empath 2.0 lead to the longest word lists, with a mean length of over 1,000 words. The average length of the word lists from LIWC is 417 words, therefore FastText delivers on average more than 5 times the number of words of the original lexica. In Table 5 of the Supplementary Materials we include the percentage of word lists for which it was possible to compute an expansion of the word list in at least one repetition of the 50 random drawing of seed words. All the methods manage to expand all of the 73 word lists from LIWC apart from WordNet, which expands only 66 thematic lexica.

**Fig. 3:**
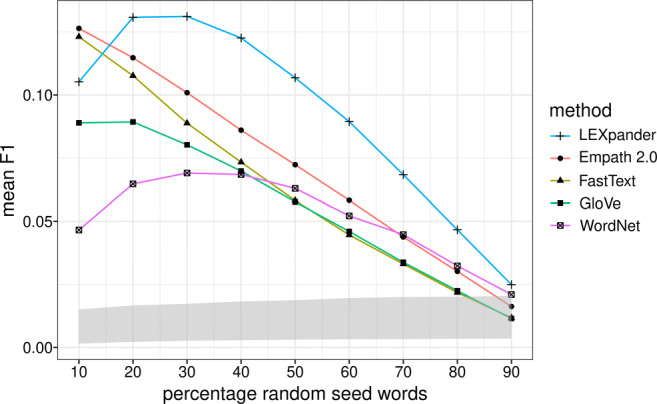
Mean of the *F*_1_ scores of the expansion of seed words chosen at random from the English 2015 version of LIWC as a function of the percentage of seed words chosen. The mean is computed on the 73 different thematic categories. The *grey area* represents the baseline as the maximum and minimum mean *F*_1_ of the random baseline methods

The results of Table 2 are relative to an initial set of seed words of 30% of the LIWC word lists. In the following, we analyze the dependence of the *F*_1_ value on the percentage of seed words chosen, as represented in Fig. 3. We also consider the values of the random baseline methods as a shaded area whose borders correspond to the minimum and maximum of the mean *F*_1_ scores relative to all the baseline methods.

In Fig. 3, we see that LEXpander achieves the best *F*_1_ in the task with the English word lists when considering at least 20% random words as seed words. With 10% of seed words, Empath 2.0 and FastText have slightly better results than LEXpander and with 90% of seed words all the methods yield very similar performances. Therefore, LEXpander proves to be consistently the best method for the expansion of word lists for many size ranges. We also see that the fewer words are needed to recover the original LIWC word lists, i.e., the higher the percentage of the random selection is, the more the random baseline methods outperform the actual lexicon expansion models. Additionally, the mean *F*_1_ of LEXpander shows a steep increase between 10% and 20% of seed words. This is probably because of the higher precision of the method which counteracts the decrease of recall when increasing the number of seed words. However, the trends in precision and recall are not substantially different from the ones of the other methods, as reported in Fig. 1 of the Supplementary Materials.

The previous results illustrate the performance of the lexicon expansion methods when dealing with a random subset of the word lists from the English LIWC. In order to analyze the dependence of the quality of the expanded word lists on the choice of the seed words, we perform the expansion of a manual selection of words from the five emotional categories of LIWC, called EVs (Vine et al., 2020). Similarly to the previous case, we find that LEXpander achieves the best precision and *F*_1_, while FastText the best recall. The full table of results for this case is reported in Table 2 of the Supplementary Materials, while a concise version constitutes the left side of Table 3. In contrast to the previous experiment, when expanding the EVs all methods achieve 100% coverage of the word lists, that is, it was always possible to compute an expansion of the EVs.

**Table 3:** Dependence of the performance of the lexicon expansion algorithms on the mode of choice of the seed words: at random or chosen by experts (EVs)

Method	EVs as seed words			Random seed words		
	prec	rec	*F*_1_	prec	rec	*F*_1_
LEXpander	**0.16**	0.10	**0.12**	**0.16** (0.02)	0.15 (0.01)	**0.15** (0.01)
WordNet^a^	0.11	0.06	0.08	0.12 (0.02)	0.08 (0.01)	0.09 (0.01)
Empath 2.0^b^	0.07	0.29	0.11	0.07 (0.00)	0.34 (0.01)	0.12 (0.00)
FastText^c^	0.06	**0.34**	0.10	0.07 (0.00)	**0.40** (0.01)	0.11 (0.01)
GloVe^d^	0.07	0.03	0.04	0.06 (0.01)	0.04 (0.01)	0.04 (0.01)

Mean of precision, recall and *F*_1_ on the five emotional categories either choosing the seed words at random from the relative LIWC dictionaries or manually by experts (EVs). The standard deviation on 50 repetitions of the random choice of seed words is reported in between parentheses. We control for the length of the seed words. The best results are highlighted in boldface

^1^ Miller (1995)

^2^ Bojanowski et al., (2017), Fast et al., (2016a)

^3^ Bojanowski et al., (2017)

^4^ Pennington et al., (2014)

In Table 3 we compare the performance of the methods when choosing the seed words at random from LIWC and when using a well-thought-out set of words to generate the final lexicon, the EVs. We consider as seed words exactly the same number of words for both cases, that is we control for the number of seed words.

In Table 3, we see that precision, recall and *F*_1_ values are always higher or equal when taking a random sample of words from LIWC than when expanding a selection of words chosen by experts. This might be due to the fact that we repeat the random choice of seed words from LIWC 50 times, averaging the estimates for precision, recall and *F*_1_. Therefore, even if one random subset of seed words is not fully representative for the theme of the word list to reconstruct, it might be balanced by the other random choices. However, the standard deviation relative to the means of precision, recall and *F*_1_ on the 50 repetitions show that the variability in the results is minimal. One alternative explanation for the observation is that the EVs were not intended to be used to reconstruct the original LIWC. Rather, the aim of their creation was to quantify the vocabulary width of people with respect to emotions. Therefore, they might collect very frequent words, while the original LIWC word lists might have a better distribution with respect to word frequency. Note that, even when the seed words were chosen at random, they were anyways selected from a thematic set of words, that is words that convey a specific meaning. Thus, this comparison does not prove that the seed words do not have to be relevant to the desired topic, but that they do not necessarily have to be the most fitting and representative ones.

We also consider the union and intersection of the expanded word lists in order to determine whether a combination of the lexica leads to better results. We find that, when expanding the EVs, the union model scores a precision value of 0.15 and a recall value of 0.04, thus leading to a *F*_1_ score of 0.06. The intersection yields to a very high precision (0.75), but the recall is 0, thus the *F*_1_ score relative to the method is 0. Therefore, the only case in which one of the combinations outperform the best scoring method consists in the precision results of the intersection model. However, this method cannot compete with the single ones with respect to recall and *F*_1_. Thus, we can conclude that the intersection and union of the word lists does not yield to better results.

#### Precision study

In Table 2 of the Supplementary Materials we compute the precision, recall and *F*_1_ score of the EVs over the original LIWC resource. Since the precision is lower than 1 (in particular, 0.86), we observe that the creators of the EVs included some words that do not appear in the original vocabulary. Thus, we can conclude that it is possible to add relevant words to LIWC, that is LIWC does not cover all the words relative to a topic. As a consequence, the precision we computed when comparing the expanded and original LIWC word lists is a lower bound for its real value. In the following precision study, we analyze this difference by collecting manual annotations of the word lists generated expanding the EVs. Since the EVs are in English, the expanded word lists can be only obtained in English. We report the lower bound for precision (indicated with *) and the estimate of its true value with respect to the annotations in Table 4.

**Table 4:** Precision study of the lexicon expansion methods when using the EVs as seed words

Method	Precision*		Precision	
	Negative	Positive	Negative	Positive
LEXpander	**0.21**	**0.20**	**0.64** [0.61,0.67]	**0.43** [0.40,0.47]
WordNet^a^	0.15	0.11	0.63 [0.60,0.67]	0.41 [0.40,0.47]
Empath 2.0^b^	0.13	0.10	0.47 [0.41,0.52]	0.35 [0.30,0.40]
FastText^c^	0.10	0.09	0.41 [0.36,0.47]	0.28 [0.23,0.33]
GloVe^d^	0.11	0.10	0.25 [0.21,0.30]	0.18 [0.15,0.21]

Comparison of the lower bound for precision (indicated with *) with the precision value adjusted according to the annotations of raters. We include 95% confidence intervals for the estimate for true precision. In bold, the best results for each computation are reported.

^1^ Miller (1995)

^2^ Bojanowski et al., (2017), Fast et al., (2016a)

^3^ Bojanowski et al., (2017)

^4^ Pennington et al., (2014)

From Table 4, we see that LEXpander achieves the highest precision value for both positive and negative word lists, as also highlighted in previous results (see Table 2). However, the estimated real precision of LEXpander is not statistically different from the one of WordNet, and the two methods perform significantly better than the other models. Therefore, methods based on word networks outperform the ones constructed on word embeddings with regard to the precision of the expansion of lexica. However, the recall score of WordNet is markedly lower than the one of LEXpander, therefore we can assume that the latter continues to score the best *F*_1_ value.

The adjusted precision reported in Table 4 is always at least 1.8 times higher than the lower bound for precision, thus corroborating the idea that the precision we could compute given the word lists from LIWC was only a lower bound. Moreover, the correlation between the lower bound and the adjusted precision values is 0.71 (*p* = 0.02). It is interesting to observe that a low value for precision does not always imply a low value in the adjusted precision: for example, in the case of the positive emotion category, methods with an estimated value for precision smaller than 0.12 yield to an estimated true value between 0.18 (GloVe) and 0.41 (WordNet). As a consequence of the new estimates for precision, we can conclude that also the values estimated for *F*_1_ in the previous tasks are lower bounds and close annotation tasks like this reveal that the true performance is higher.

#### Convergent validity in text analysis

**Fig. 4:**
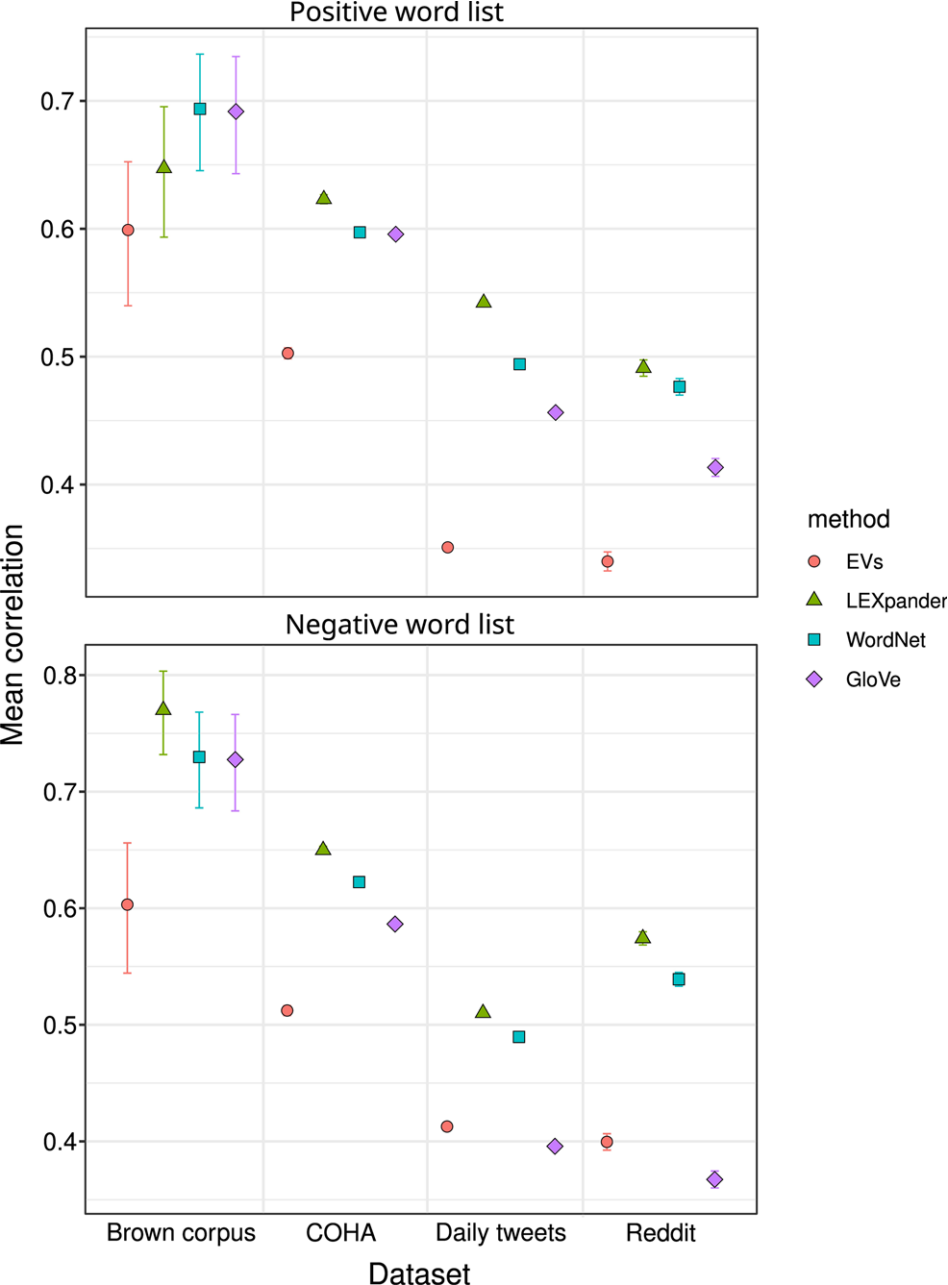
Correlation of the frequency of words in the positive (*top*) and negative (*bottom*) expanded word lists from the EVs and the ones from LIWC in texts from different datasets. The performances of the EVs is considered as a baseline. The *bars* indicate the 95% confidence intervals. In some cases, error bars are narrower than point size

As a last test, we perform a text analysis validation task on long and short English texts from online and offline communication. We compare the frequencies computed using the expanded word lists with the ones of the EVs and the original LIWC word lists on each dataset. We consider the annotated word lists from the precision study, which were expanded from the positive and negative EVs using LEXpander, GloVe and WordNet and report the correlations in Fig. 4.

In the text analysis exercise, we see that LEXpander always achieves best or tied with the best correlation on all the datasets. In particular, LEXpander, WordNet and GloVe yield statistically indistinguishable results from the ones of the EVs on the Brown corpus for the positive lexicon. With respect to negative sentiment, the three models are indistinguishable but significantly outperform the baseline given by EVs. On the other datasets, namely COHA, Reddit and the tweets published in one day, LEXpander achieves the best correlation with the positive and negative word lists from LIWC, outperforming also the EVs.

### LIWC 2007 in German

In this section, we consider the performance evaluation of the lexicon expansion algorithms on the German version of LIWC from 2007.

#### Performance evaluation

We test the performance of LEXpander and the other lexicon expansion algorithms when dealing with seed words in German. Similarly to the case of the English word lists, we expand a random selection of seed words of the German version of LIWC from 2007. In Table 5 we report the results relative to a 30% random selection of words.

**Table 5:** Results of the lexicon expansion task with a random selection of 30% words from the lexica of the German LIWC

Method	Precision		Recall		*F*_1_		Mean size
	Mean	bl	Mean	bl	Mean	bl	
LEXpander	**0.20**	0.01	0.11	0.01	**0.14**	0.01	468
OdeNet^a^	0.03	0.00	0.00	0.00	0.00	0.00	170
Empath 2.0^b^	0.03	0.01	0.14	0.02	0.04	0.01	1,905
FastText^c^	0.03	0.01	**0.16**	0.03	0.04	0.01	2,350
GloVe^d^	0.05	0.01	0.13	0.02	0.05	0.01	722

Precision, recall and *F*_1_ of the word list retrieved with 30% of seed word chosen at random versus the original word lists from the German LIWC. We report the performance of the relative random baseline method (bl) and the mean size of the expanded word lists. In bold are highlighted the best performances

^a^ Siegel and Bond (2021)

^b^ Bojanowski et al., (2017), Fast et al., (2016a)

^c^ Bojanowski et al., (2017)

^d^ Pennington et al., (2014)

In Table 5 we see that LEXpander yields to the best values for precision and *F*_1_ in German, while FastText achieves the best recall, thus confirming the trend already observed with English in Table 2: LEXpander features the best precision and the best trade-off between recall and precision overall. We observe that in general the mean size of the final word lists (see Table 4 of the Supplementary Materials) is larger than the one obtained in the English setting. This is probably a result of the fact that German has more word inflections than English. For example, while the word ‘friend’ in English can only be inflected with the plural ‘friends’, in German the base word ‘Freund’ can be inflected in several ways, as for example ‘Freundin’ (feminine singular), ‘Freundinnen’ and ‘Freunde’ (respectively feminine and masculine plural), ‘Freunden’ (masculine dative plural), ‘Freunds’ and ‘Freundes’ (masculine genitive singular) and so on. Also in this case, we find that FastText and Empath 2.0 deliver the largest word lists, while OdeNet is characterized by the shortest outcomes.

**Fig. 5:**
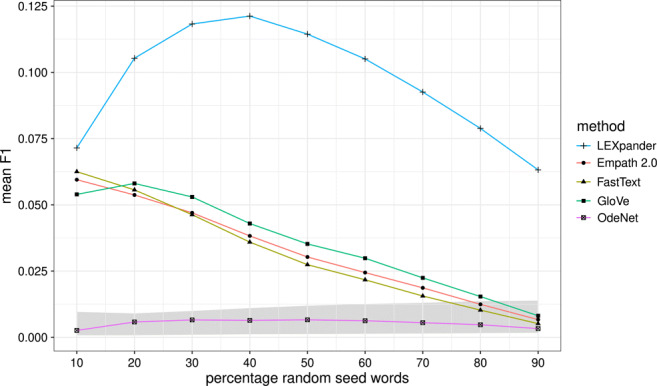
Mean of the *F*_1_ scores of the expansion of the German LIWC as a function of the percentage of words chosen at random from the original lexicon. The *grey area* represents the maximum and minimum of the random baseline methods

Similarly to the experiment with English word lists, also in the German case we consider the value of *F*_1_ as a variable of the number of seed words, as represented in Fig. 5.

Figure 5 shows that LEXpander reaches the best *F*_1_ for any size of seed words chosen. Moreover, the *F*_1_ results and the relative difference in performance of the methods increases the more seed words are considered until 40% of seed words, and then decreases, while the results of the random baseline methods increase steadily. We observed a similar pattern in the case of the English LIWC (see Fig. 3), with the increase stopping at around 20% of seed words. This happens because, the higher the percentage of seed words given as input, the fewer words have to be added to the expanded word list in order to recover the original one. We also observe that OdeNet delivers worse results than the random baseline method, i.e., a random model achieves better *F*_1_ than OdeNet. This is probably due to the limited size of the OdeNet network as hinted by the number of word lists the method manages to expand: 27 out of the 68 word lists in the German LIWC. All the other methods expand more than 60 word lists and LEXpander, Empath 2.0 and FastText achieve the highest number of expanded word lists: 64 out of 68. These results are reported in Table 5 of the Supplementary Materials.

## Discussion

In this paper, we present a new lexicon expansion algorithm, LEXpander, and compare its performance in a benchmark including different automatic lexicon expansion algorithms. We show that LEXpander achieves the best precision and *F*_1_ in the lexicon expansion tasks in two linguistic settings. Moreover, it is best or tie with the best in an English text analysis exercise. LEXpander is an open-source method available as a web tool (https://annadinatale.shinyapps.io/lexpander_app/). Moreover, the word lists expanded from the EVs are shared on the GitHub page (https://github.com/AnnaDiNatale/LEXpander), as well as the code realized along with the present paper. LEXpander is a lexicon expansion algorithm based on a linguistic concept, colexification, and the present publication shows the usefulness of bridging linguistic theory and NLP applications. Incorporating linguistic theory can provide novel, interpretable models, which give insights into phenomena rather than fitting statistical features of texts with black box algorithms.

Our work also shows that some linguistic ideas can solve the problem of under-performing methods for languages different from English. Indeed, when deploying concepts that are supra-lingual or independent from language, as colexification networks, to build linguistic methods, such methods can easily be applied to a plethora of languages. Indeed, in this paper we used the same architecture and the same underlying data structure (the colexification network) to solve the problem of lexicon expansion in two different languages achieving good performances in both linguistic settings. In our view, independence from language does not mean that the method yields to the same results in every language; rather that the same method can be used without adjustments to expand lexica in different languages. Moreover, the evidence that the quality of the results of LEXpander in the German setting is more pronounced than the one in English proves that the existing methods are lacking when applied to languages different from English. This is because all the other word list expansion methods considered here were developed and validated taking into account only English. By making use of the idea of colexification, which deals with supra-lingual concepts, we show the potential applications enabled by this property of the method.

In addition to this, we find that lexicon expansion methods based on networks outperform the ones based on word embeddings in terms of precision and *F*_1_. We also prove that the union of the expanded word lists does not yield to better results. Indeed, the union of word lists contributes to raising the recall at the expense of precision, thus resulting in a low estimate for *F*_1_. However, the high recall can benefit applications to a different range of problems, as for example in the area of text mining. In particular, Bozarth and Budak (2022) highlight that when considering the amount of relevant tweets that keyword expansion algorithms can retrieve, the union of expanded lexica leads to better results than the ones achieved by the single word lists.

While LEXpander and other lexicon expansion methods offer an easy way to improve word lists, we do not recommend using them without some degree of manual inspection and filtering. Such selection should be performed taking into account the application and the type of language of the texts considered. For example, in the text analysis exercise we showed that the positive and negative word lists obtained with LEXpander have the highest correlation with LIWC on all the dataset considered. However, the annotation of the expanded word lists had been executed without the aim of performing such an application. Therefore, we think that the performance of all the methods would have been better if the cleaning process would have been completed with a specific dataset in mind.

Moreover, there are some cases where researchers might want to use other methods than LEXpander, especially when focusing on niche domains and contexts. In particular, LEXpander does not allow to explore novel ways of usage of language in a specific linguistic environment, as word embeddings do when trained on novel corpora. Therefore, when exploring the language used in a medium to find patterns never analyzed before, as in Balsamo et al., (2021), word embeddings seem to offer a better alternative. On the contrary, LEXpander is the method to use in the pre-processing of data, as for example when selecting text instances relative to a topic or when brainstorming for the creation of word lists related to general topics. Moreover, the supra-lingual feature of LEXpander makes it the preferable choice for these tasks when considering languages with lower resources compared to English.

LEXpander is a resource that can be used both when brainstorming and compiling lexica and when doing text analysis after an adequate cleaning. Future work with LEXpander may include the analysis of psychological phenomena with the help of this resource. For example, the dictionary from the Moral Foundation Theory (Graham, Haidt, & Nosek, 2009; Graham et al.,, 2013) may be expanded for better capturing signals in texts. Another application may rely on the creation of novel word lists, as for example ones intended to test new concepts from psychology or sociology, as the ideas of loose and tight cultures in Jackson, Gelfand, De and Fox (2019).

To summarize, we find that LEXpander combines a high coverage of the thematic categories and the best trade-off between precision and recall in the task of expanding a word list both in English and German. The absolute values of the performance might be deceivingly low: the best method, LEXpander, achieves a *F*_1_ score of 0.12 when expanding the EVs. However, this value represents only a lower bound for the actual *F*_1_ score, as proven by the precision study. Indeed, we prove that the precision value we compute is a lower bound and that, in the case of LEXpander, the real precision value is at least two times higher than its lower bound.

## Conclusions

In this paper, we introduce a novel lexicon expansion algorithm, LEXpander. LEXpander implements a method based on a colexification network, that is a multilingual semantic network. We test the performance of LEXpander on various lexicon expansion tasks, comparing it to other widely used lexicon expansion algorithms, including methods based on the GloVe and FastText word embeddings, and algorithms deploying semantic networks, as WordNet and its German counterpart, OdeNet. We find that LEXpander is the best option when focusing on precision or *F*_1_ both in English and German. The German experiment shows the performance of the method in a non-English setting, but the tool can be applied to all the other languages featured in the colexification network, which amount to 19 languages belonging to four different families.

Even if LIWC might seem the best method for some tasks, it is not open source, therefore an alternative method might be more widely used. A freely available tool is Empath (Fast et al., 2016a), often deployed in research thanks to its ease of use. However, Empath is now outdated and delivers very short expanded word lists. LEXpander is a free, open-source multilingual tool that can be found in a GitHub repository (https://github.com/AnnaDiNatale/LEXpander/releases/tag/v1.0.0https://github.com/AnnaDiNatale/LEXpander/releases/tag/v1.0.0 ) and can be used through an interactive page (https://annadinatale.shinyapps.io/lexpander_app/). Moreover, we share the word lists obtained from the expansion of the EVs on GitHub (https://github.com/AnnaDiNatale/LEXpander/tree/main/expanded_wordlists/Annotated), which provide a resource comparable to the emotional LIWC word lists without using any LIWC dictionary data. These resources are freely available for download, with the aim of supporting future research on text analysis methods and their applications.

### Electronic supplementary material

Below is the link to the electronic supplementary material.

(PDF 267 KB)

## Data Availability

The datasets created and/or analyzed are available in the GitHub repository, https://doi.org/https://github.com/AnnaDiNatale/LEXpander/releases/tag/v1.0.0, or on Zenodo, 10.5281/zenodo.7377095, with the exception of LIWC word lists, which can be purchased by any researcher from https://www.liwc.app/.

## Footnotes

1 This can also happen in word embedding models, thus we report this for all methods in our benchmark.

2 We decided to exclude the undifferentiated negative emotion vocabulary of the EVs from this study because it does not match any of the original thematic word list of LIWC.

## Code availability

Codes for reproducing our results are available in the GitHub repository, https://github.com/AnnaDiNatale/LEXpander/releases/tag/v1.0.0and on Zenodo 10.5281/zenodo.7377095. LEXpander is also available as an online tool for word lists expansion: https://annadinatale.shinyapps.io/lexpander_app/.
