# Supplementary material for: LEXpander: Applying colexification networks to automated lexicon expansion
Source: Behav Res Methods. 2023 Mar 10;56(2):952–67. doi: 10.3758/s13428-023-02063-y (PMC10000354; doi:10.3758/s13428-023-02063-y)
Supplement: Supplementary file 1 — (PDF 267 KB) [file 13428_2023_2063_MOESM1_ESM.pdf]

LEXpander supplementary materials

Anna Di Natale, David Garcia

In the first task we expand a set of seed words into a word list. Expanding a random sample of 30% of the words in each category of the English LIWC, we find word lists with different lengths. Table 1 reports the lengths of the final word lists for the 5 emotional categories and the mean length on all the 73 categories in comparison to the length of the original lexica from LIWC.

	LIWC	LEXpander	WordNet	Empath 2.0	FastText	GloVe
Negemo	1,410	1,626	1,222	3,227	5,916	1,873
Posemo	1,052	1,966	1,839	4,019	6,977	1,613
Anx	263	428	331	3,170	3,681	311
Anger	455	656	668	3,020	4,201	516
Sad	258	464	327	2,862	3,333	440
mean	417	614	525	1,293	2,252	773

Table 1: Mean length of the expanded word lists with 30% random words from the English LIWC as seed words. We report the mean lengths of the emotional categories on 50 repetitions and the mean over all the categories.

Table 1 shows that the word lists obtained with FastText and Empath 2.0 are the longest, consisting on average of nearly 3 times more words than the original lexicon.

We then consider the reason for the steep increase in performance of LEXpander between 10% and 20% of the seed words. In Figure 1 we plot the precision (top) and the recall (bottom) of all the methods as a function of the percentage of random seed words.

As we can see, the trends are similar (the precision decreases with length of the seed words, while the recall has a quadratic trend and reaches a maximum before decreasing towards the end of the scale). The performance of LEXpander is characterised by a steeper increase in recall which lasts longer than the one of the other methods. This might be the reason why LEXpander shows an increase in mean F_1 between 10% and 20% of random seed words.

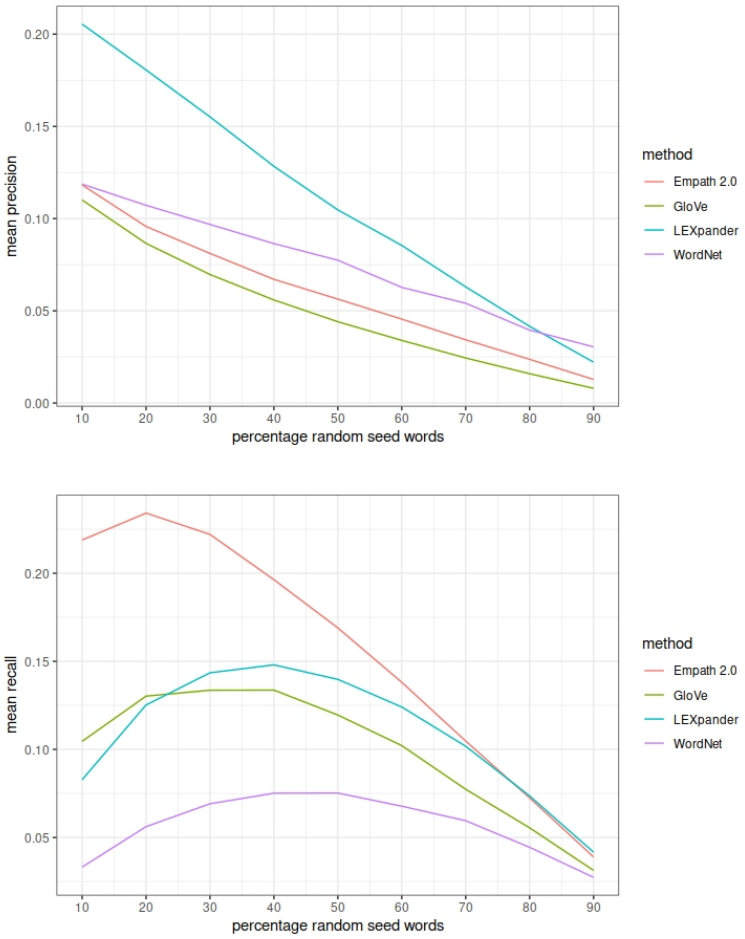

Figure 1: Mean precision (top) and mean recall (bottom) of the lexicon expansion methods as a function of the percentage of seed words selected. The trends for all the methods are similar, but LEXpander reaches a higher precision, which seems to drive the mean F_1 of the method.

Table 2: Results of the expansion of the EVs.

Method	Precision		Recall		F_1		mean
	mean	bl	mean	bl	mean	bl	size
Original EVs	0.86	-	0.19	-	0.30	-	132
LEXpander	**0.16**	0.02	0.10	0.01	**0.12**	0.02	570
WordNet	0.11	0.00	0.06	0.00	0.08	0.00	492
Empath 2.0	0.07	0.02	0.29	0.07	0.11	0.03	2,702
FastText	0.06	0.02	**0.34**	0.10	0.10	0.03	3,684
GloVe	0.07	0.01	0.03	0.01	0.04	0.01	419

Mean of precision, recall and F_1 of the expansion of the EVs in comparison to the relative word lists of LIWC. The results of the baseline models (bl) are also reported. The mean of the performances is computed on the five emotional word lists. The best results are indicated with boldface. In this case, we also report the comparison of the original EV wordlists with the lexica from LIWC (first row).

In the comparison between the EVs and LIWC we observe that the precision is lower than 1, which would have been expected if the EVs would have been created choosing only words from LIWC. Therefore, when creating the EVs, researchers added some words which were not present in the original LIWC word lists. This hints to the fact that LIWC is not the most extensive source, at least for emotional word lists.

In Table 3 we report the length of the word lists obtained expanding the EVs. Also in this case, FastText and Empath 2.0 yield to the largest lexica.

length	EVs	LEXpander	WordNet	Empath2.0	FastText	GloVe
Negemo	276	1,068	979	3,325	5,288	672
Posemo	172	815	685	2,723	3,835	878
AnxFear	62	241	194	2,579	3,312	186
Anger	55	285	279	2,417	2,960	128
Sad	95	443	325	2,468	3,023	229
mean	132	570	492	2,702	3,683	419

Table 3: Length of the expanded word lists using the EVs as seed words. The mean is computed on the 5 emotional categories.

We then expand a random selection of seed words from the German LIWC in order to test whether the methods yield to comparable results in a different linguistic setting. The mean length of the word lists obtained with 30% of seed words is reported in Table 4.

Also in this case, both FastText and Empath 2.0 output large word lists, but the discrepancy from the other methods is less dramatic than in the case

of English. On the opposite, OdeNet yields to the shortest word lists and does not manage to expand the word list for Anger.

	LIWC deu	LEXpander	OdeNet	Empath 2.0	FastText	GloVe
Negemo	2,130	1,291	676	4,900	8,815	1,475
Posemo	1,576	1,134	503	4,656	7,394	1,791
Anx	276	197	94	3,138	2,948	204
Anger	570	349	169	3,123	3,620	361
Sad	462	294	147	3,728	3,619	464
mean	528	346	170	1,905	2,744	722

Table 4: Length of the expanded word lists with 30% random words from the German LIWC as seed words. We report the mean length of the emotional categories over 50 repetitions of the random choice and the mean of the lengths over all the categories.

Some of the methods might not be able to extend some word lists. Indeed, in the case of methods based on networks, it might not be possible to match the seed words on the network or those words might be part of an unconnected component. In the case of word embeddings, the seed words might be isolated from other words. In Table 5 we report the percentage of wordlists which could be expanded with each method. In the case of the random selection of seed words (LIWC in English, LIWC en, and LIWC in German, LIWC deu) we consider the percentage of wordlists which could be expanded in at least one of the 50 random sampling of the seed words.

	EVs	LIWC en	LIWC deu
LEXpander	100%	100%	94%
WordNet	100%	92%	-
OdeNet	-	-	40%
Empath 2.0	100%	100%	94%
FastText	100%	100%	94%
GloVe	100%	100%	88%

Table 5: Percentage of the word lists for which it was possible to compute an expanded version. In the case of LIWC in English and German, the case with 30% random words is considered. Some methods could not be applied to word lists in one language. We use the symbol - to indicate such cases.

From Table 5 we see that the EVs could always be expanded by all the methods. On the opposite, the selections of 30% random words from the LIWC lexica in English and German show a different score. In particular, WordNet and OdeNet have the lowest score respectively on LIWC in English and in German. Apart from WordNet, all other methods could recover 100% of the wordlists from the English LIWC, while none of them has the same performance on the

German lexica, where the highest percentage, 94%, is reached by LEXpander, Empath 2.0 and FastText.
